# S74. EXPLORING PARTICIPANT-LEVEL TRAJECTORIES OF COGNITIVE PERFORMANCE AMONG PATIENTS WITH SCHIZOPHRENIA IN A MULTI-NATIONAL TRIAL

**DOI:** 10.1093/schbul/sby018.861

**Published:** 2018-04-01

**Authors:** Kiri Granger, Jack Cotter, Elizabeth Baker, John Evenden, Jennifer Barnett, Michael Sand

**Affiliations:** 1 Cambridge Cognition; 2 Boehringer Ingelheim Pharma GmbH & Co. KG

## Abstract

**Background:**

It remains unclear whether the lack of clinical trial success and drug approval for cognitive impairment associated with schizophrenia (CIAS) is due to compounds being ineffective, or whether trial methodology itself has been a limiting factor in successfully demonstrating the efficacy of these agents. Schizophrenia is a heterogeneous disorder and whilst cognitive deficits are a core feature, the profile and degree of neuropsychological impairment can vary across patients. Though most individuals with schizophrenia exhibit some general cognitive impairment compared to antecedent expectations, such as premorbid intelligence, up to a quarter display cognitive performance in the ‘normal’ range. This may pose a problem for pro-cognitive drug trials in this population given that it potentially inflates baseline scores and reduces the scope to see improvement between treatment and placebo groups. In order to examine this potential issue, we investigated participant-level trajectories of cognitive performance among patients with schizophrenia enrolled in a multi-national, phase II clinical trial.

**Methods:**

We conducted a post-hoc analysis of existing trial data from 463 patients with schizophrenia who participated in a randomized, double-blind, placebo-controlled trial. Patients met established diagnosis for schizophrenia (DSM-5), were clinically stable (non-acute) and had no more than moderate severity ratings on the Positive and Negative Syndrome Scale (PANSS). During the trial, participants completed two different neurocognitive test batteries, the Cambridge Neuropsychological Test Automated Battery (CANTAB) and the MATRICS Consensus Cognitive Battery (MCCB), at 4 separate time points (screening, baseline, week 6 & week 12). Participant data were pooled across placebo and treatment groups to explore trajectories of cognitive performance, at the participant-level, across the course of the study.

**Results:**

Linear mixed model analyses revealed that participants who performed within the ‘normal range’ at screening on cognitive tasks as measured by CANTAB, continued to perform well at baseline, week 6 and week 12, showing no significant change in their performance. By contrast, participants who performed below the normal range at screening, showed a significant improvement in their test performance across the remainder of the study. When compared in the context of MCCB, those participants who performed a standard deviation (SD) above the MCCB normative mean at screening, were also the participants who performed within the normal range on CANTAB. Approximately 25% of the overall sample were performing within a clinically normal cognitive range at screening.

**Discussion:**

Substantial variability was evident in cognitive performance among the current sample of patients with schizophrenia. We identified a subsample of patients whose performance fell within a clinically normal range. Cognitive improvement was observed only in those who exhibited a deficit at screening, bringing into question whether the inclusion of unimpaired patients in clinical trials increases the risk of ceiling effects and minimizes chance to see change. Further analyses will determine the interaction between different cognitive trajectories and the treatment arms included in this trial to explore whether there are individuals with a particular cognitive profile who are most likely to respond to treatment. This has potentially important methodological implications in the search to find a drug to treat CIAS.

